# Correcting for Spectral Cross-Talk in Dual-Color Fluorescence Cross-Correlation Spectroscopy

**DOI:** 10.1002/cphc.201100801

**Published:** 2012-02-16

**Authors:** Kirsten Bacia, Zdeněk Petrášek, Petra Schwille

**Affiliations:** [a] HALOmem, University of HalleKurt-Mothes-Str. 3, 06120 Halle (Germany), Fax: (+49) 345-55-27408; [b] Institute of Biophysics, BIOTECTU Dresden, Tatzberg 47–51, 01307 Dresden (Germany)

**Keywords:** binding analysis, biophysics, fluorescence, fluorescence correlation spectroscopy, fluorescence spectroscopy

## Abstract

Dual-color fluorescence cross-correlation spectroscopy (dcFCCS) allows one to quantitatively assess the interactions of mobile molecules labeled with distinct fluorophores. The technique is widely applied to both reconstituted and live-cell biological systems. A major drawback of dcFCCS is the risk of an artifactual false-positive or overestimated cross-correlation amplitude arising from spectral cross-talk. Cross-talk can be reduced or prevented by fast alternating excitation, but the technology is not easily implemented in standard commercial setups. An experimental strategy is devised that does not require specialized hardware and software for recognizing and correcting for cross-talk in standard dcFCCS. The dependence of the cross-talk on particle concentrations and brightnesses is quantitatively confirmed. Moreover, it is straightforward to quantitatively correct for cross-talk using quickly accessible parameters, that is, the measured (apparent) fluorescence count rates and correlation amplitudes. Only the bleed-through ratio needs to be determined in a calibration measurement. Finally, the limitations of cross-talk correction and its influence on experimental error are explored.

## 1. Introduction

Fluorescence correlation spectroscopy (FCS) has become a standard technique for characterizing the dynamic behavior of biomolecules in a variety of systems, ranging from aqueous buffer solutions and lipid membranes to living cells and organisms. Dual-color fluorescence cross-correlation spectroscopy (dcFCCS), a variant of FCS that exploits the cross-correlation *G*_rg_(*τ*) between two spectrally separated detection channels, is particularly powerful for analyzing molecular interactions between different species^1^, ^2^ and is now widely used for biochemical studies in situ.^3^–^8^ In the ideal case when the cross-correlation is neither diminished by incomplete labeling or detection volume overlap^9^ nor enlarged by spectral cross-talk,^10^, ^11^ degrees of binding are determined from the relative cross-correlation amplitudes: the number of double-labeled (“bound”) particles *N*^RG^ relative to the total number of particles carrying a red label *N*^R^ (including double-labeled ones) equals the amplitude of the cross-correlation curve *X*=*G*_rg_(0) relative to the amplitude of the green autocorrelation curve, *G*_g_,^10^ and vice versa [Eq. (1)]


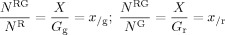
(1)

where the fluorescence cross-correlation function is defined by [Eq. (2)]


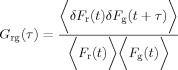
(2)

The convenience of commercial FCS setups has greatly fostered applications of dcFCCS to cell biological questions.^12^–^16^ Herein, we revisit the treatment of the cross-talk artifact, which is a major obstacle during the implementation of dcFCCS in standard commercial setups.

**Figure 1:**
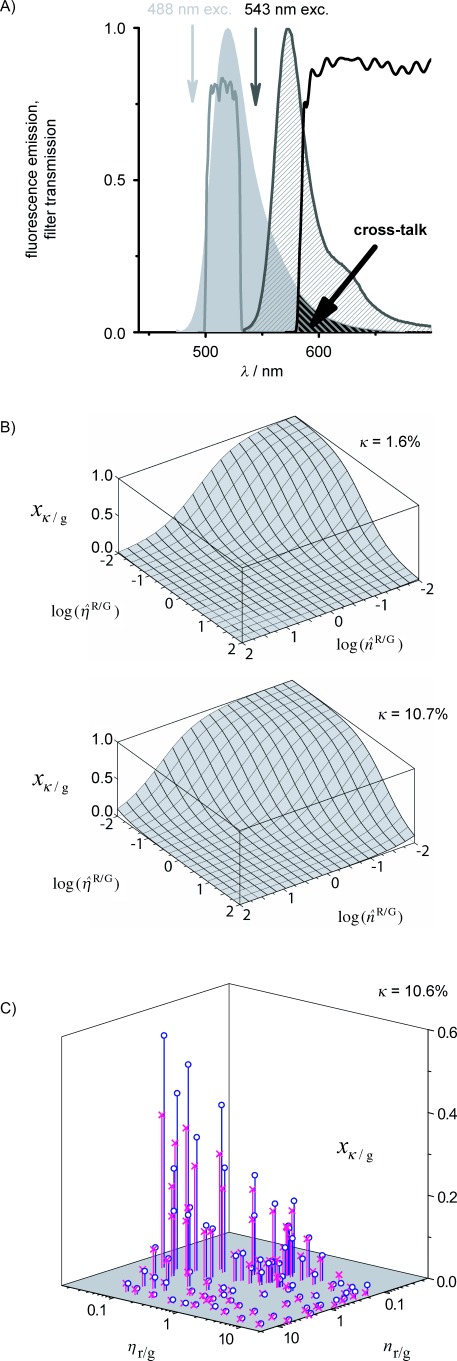
The extent of the cross-correlation artifact caused by spectral cross-talk depends on particle brightnesses and concentrations. A) Spectral bleed-through. The emission spectra of the dyes (filled gray: Alexa 488, hatched: Alexa 546) are not perfectly separated by the emission filters (—). The arrow points to the overlap of the green emission with the transmission of the filter in the red detection channel, which is the cause of the bleed-through. The bleed-through parameter *κ* for given spectral characteristics of the setup and dyes needs to be determined experimentally [Eq. (3)]. B) Simulation of the effect of the bleed-through parameter *κ* on the relative cross-correlation amplitude. The plots show the calculated amplitude of the cross-correlation relative to the green autocorrelation for a mixture of green and red molecules in the absence of truly double-labeled species. The entirely artifactual relative cross-correlation 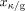 is depicted as a function of the brightness ratio 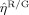 and number ratio 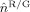 of the red and green species according to Equation (34c). The upper plot was calculated for a very small bleed-through ratio (*κ*=1.6 %, Alexa 488 and Cy5), the lower plot for a larger bleed-through ratio (*κ*=10.7 %, Alexa 488 and Alexa 546). C) Attempt to approximate the artifactual relative cross-correlation 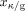 by substituting apparent brightnesses and numbers ( 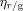, 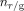) for true brightnesses and numbers ( 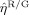, 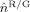) in Equation (34c). The circles show the measured artifactual relative cross-correlations, the crosses the predictions of the cross-talk artifact. A rather large discrepancy between prediction and measurement is obtained. Equating true and apparent parameter values should hence be avoided.

The stringent requirements on fluorophore properties for use in FCS, such as exceptional brightness, photostability, biocompatibility, and spectral compatibility with the setup, limit the choice of dyes available for labeling. As a consequence, it is next to impossible to achieve a full spectral separation of different fluorescent species.^17^ This is particularly true for intracellular applications with genetically encoded probes, where the green and red fluorescent proteins suited for FCS (e.g. eGFP and mCherry) preclude as good a spectral separation as can be achieved with chemical dyes (e.g. Alexa 488 and Cy5). As a consequence, the contribution to the cross-correlation amplitude from cross-talk (bleed-through) of one dye into the other channel (Figure 1 A) can create a serious artifact, depending on the conditions of the measurement.^11^, ^18^–^20^ We have developed experimental strategies for minimizing and correcting the artifactual contribution to the cross-correlation measurement caused by this bleed-through. In the following, for simplicity, the dye pair and the detectors will be referred to as “green” and “red” and the corresponding excitation lines as “blue” and “orange”, thereby denoting their relative positions on the wavelength axis, without a limitation to these particular colors.

The extent of the cross-talk-induced artifact depends, albeit not exclusively, on the ratio of the fluorescence obtained from a fluorophore in the “wrong” channel, compared to the fluorescence obtained from the same fluorophore in the “correct” channel. This ratio is denoted as the bleed-through ratio 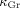 of the Green dye to the red channel, and 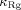 of the Red dye to the green channel (see Table 1 for an overview of parameters). The values of the bleed-through ratios 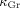 and 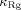 are primarily governed by the choice of dyes (spectral overlap) and the choice of filters. If the separation of the dyes is of the quality shown in Figure 1 (Alexa Fluor 488 and Alexa Fluor 546 and typical filters) or better (e.g. eGFP and mCherry, or Alexa Fluor 488 and Cy5 and suitable filters), only cross-talk from the green dye into the red channel needs to be taken into account ( 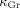
=*κ*, 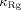
=0*)*.

**Table 1:** Definitions of the parameters employed[a]

Absolute quantities	
*G*_g_	apparent correlation amplitude in the green channel
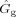	true correlation amplitude in the green channel
*X*	apparent cross-correlation amplitude
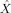	true cross-correlation amplitude
*X*_*κ*_	artifactual cross-correlation amplitude due to cross-talk
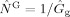	total number of Green-labeled particles in the detection volume (including double-labeled particles)
*N*^G^=1/*G*_g_	apparent total number of Green-labeled particles
*V*_eff(g)_	detection volume of the green channel
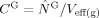	total concentration of Green-labeled particles
*F*_g_(*t*)	apparent time-dependent fluorescence count rate in the green channel
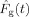	true time-dependent fluorescence count rate (i.e. in the absence of cross-talk or after cross-talk correction)
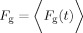	apparent green fluorescence count rate (time-averaged)
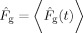	true green fluorescence count rate (time-averaged in the absence of cross-talk or after cross-talk correction)
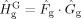	true brightness^[b]^ of a Green-labeled particle in the green channel
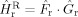	true brightness of a Red-labeled particle in the red channel
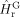	true brightness of a Green-labeled particle in the red channel
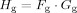	apparent brightness in the green channel

Relative quantities (denoted by small letters)	
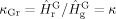	bleed-through ratio: brightness of the Green dye in the “wrong” (red) channel divided by the “correct” (green) channel
*f*=*F*_g_/*F*_r_	apparent count-rate ratio
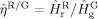	true brightness ratio: ratio of the brightnesses of the Red dye in the red channel and the Green dye in the green channel (obtained from a separate, cross-talk-free measurement)
*η*_r/g_=*H*_r_/*H*_g_	apparent brightness ratio: brightnesses are obtained directly from the cross-talk-affected measurement (*H*_r_=*F*_r_*G*_r_)
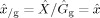	true relative cross-correlation amplitude
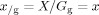	apparent relative cross-correlation amplitude
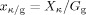	artifactual relative cross-correlation amplitude, arising from cross-talk
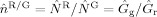	true red-to-green number ratio^[c]^
*n*_r/g_=*N*_r_/*N*_g_=*G*_g_/*G*_r_	apparent red-to-green number ratio

[a] Indices g and r refer to the green and red detection channel, respectively; superscripts G and R to Green and Red particles. The hat symbol (∧) denotes true quantities, that is, quantities that have been corrected for cross-talk or are not affected by cross-talk. All quantities are defined analogously for the green and red channels. [b] Brightness denotes the fluorescence count rate per particle. [c] The number ratio equals the concentration ratio if the detection volume is of the same size for the different channels ( 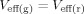). In the setup used here, the red detection volume is larger.

We restrict our discussion to this unidirectional cross-talk because it enables a more intuitive understanding, and it is encountered with the most commonly employed dye and filter combinations in dcFCCS. However, in cases of less spectral separation, bidirectional cross-talk theory needs to be applied.

The bleed-through ratio *κ*= 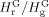 (where 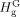 denotes the brightness of the Green dye in the green channel and 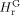 the brightness of the Green dye in the red channel) is determined from a one-time calibration measurement. Only green dye is used and the average count rate in the red channel is divided by the average count rate in the green channel. Background count rates from a dark measurement are subtracted,^11^ but were of negligible influence in our experiments [Eq. (3)]


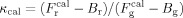
(3)

Cross-talk has an enormous potential to cause artifactual cross-correlation. If we consider a sample containing only green-labeled particles, measured with high detection efficiency and low noise, even small percentages of bleed-through result in all green molecules being detected in the red channel. In the absence of double- and red-labeled particles, the auto- and cross-correlation curves will all coincide, that is, they appear as if there was 100 % binding. In contrast, in an appropriately designed binding assay, where a sufficient number of red- or double-labeled molecules with sufficient brightness in the red are present, the red bleed-through signal from the green-labeled molecules becomes negligible.

In the following, it is demonstrated how the artifactual effect of cross-talk on the cross-correlation amplitude can be calculated from the bleed-through ratio *κ* and the apparent (i.e. measured) count rates and amplitudes. Furthermore, we show how the correction of the cross-talk-induced contribution to the cross-correlation can be evaluated.

Mathematical expressions for cross-talk correction have been derived and employed to correct dcFCCS data in several previous works. Crucially, the theoretical foundation for cross-talk correction was provided by the seminal contribution of Ricka et al.^18^ (see Experimental Section). Equations tailored to various complex experimental situations were provided, for example, by Weidemann et al.,^9^ Földes-Papp,^19^ and Hwang et al.^20^ However, a systematic test of the validity and accuracy of cross-talk correction has been missing. Herein, we show that the resulting cross-talk artifact is a function of the bleed-through value and two important ratios, namely the number ratio and the brightness ratio. In contrast to previous works, we systematically varied the number and brightness ratios and devised a way to measure the magnitude of the artifact and compare it to the magnitude of the correction term. Our three-dimensional plots illustrate the strong dependence of the cross-talk artifact on these ratios and show that cross-talk correction approaches cannot be validated using only a few scenarios. We have newly identified a critical quantity that determines the magnitude of the cross-talk artifact and the limitations in performing any corrections. Furthermore, we show that it is not necessary to determine the true, cross-talk-unaffected particle number from separate, single-color measurements, as was required in previous correction approaches.^19^, ^21^ We show that cross-talk correction based on the apparent brightness and number ratios requires fewer experimental steps and is more accurate. Finally, our cross-talk correction approach is independent of such intricacies as complex binding stoichiometries and fluorophore quenching.

In summary, the critical advantages of the approach for cross-talk assessment and correction that will be described in the following are its ease of use, its experimental validation, and the intuitive understanding gained by virtue of its simplicity.

## 2. Results and Discussion

The correct concentrations of double-labeled species from cross-correlation measurements involving cross-talk can be calculated based on all the particle brightnesses ( 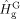, 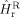, 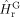, see Table 1 for parameter definitions) determined in separate measurements.^21^ In experimental systems in which samples can be rapidly and reproducibly prepared, these brightnesses are easily determinable from separate samples that contain exclusively the green- and the red-labeled species. If such separate samples cannot be prepared, the brightnesses may be assessed from the actual sample using single excitations. However, single-excitation measurements neglect the contribution of cross-excitation, as discussed below. Moreover, these separate measurements consume extra time, which is a concern with preparations of limited stability, such as live cells. Also, if laser powers are varied during an experiment, brightnesses need to be determined again. We therefore focused on how the cross-correlation artifact can be minimized by experimental design, and how the residual cross-talk can be conveniently corrected by using the data of the actual cross-correlation experiment.

### Minimizing Cross-Talk

Expressing the artifactual contribution to the cross-correlation amplitude in terms of relative quantities gives insight into how experiments can be designed to control cross-talk [Eqs. (34c), (35c)]. These quantities are the bleed-through ratio *κ*, which is constant for a fixed setup and dye spectrum, the ratio of the numbers of green and red particles ( 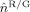), and the ratio of the brightnesses of the green and red particles in their proper channels ( 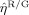). To assess the influences of these parameters on the cross-correlation, relative cross-correlation amplitudes were determined on samples that do not contain any double-labeled species. In this case, the relative cross-correlation amplitudes are entirely caused by cross-talk and are denoted by *x*_*κ*/g_ and *x*_*κ*/r_. Figure 1 B shows that the artifactual cross-correlation *x*_*κ*/g_ becomes minimized when the red-labeled molecules are in excess, and when the red-labeled molecules are brighter than the green in their respective channels. If possible, it is therefore advisable to design the experiment such that the red, and not the green, particles are in excess. Furthermore, the powers of the exciting lasers should be adjusted such that the red brightness is larger than the green brightness. As a convenient rule of thumb for small *κ*, we note that when the red versus green numbers as well as the brightnesses are balanced ( 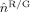
=1, 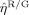
=1), the artifactual relative cross-correlation amplitudes in the absence of double-labeled species both equal *κ* (*x*_*κ*/g_ 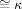 and *x*_*κ*/r_ 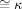; Equation (36a/b), derived in the Experimental Section).

### Cross-Talk Prediction from the Actual Measurement

**Figure 2:**
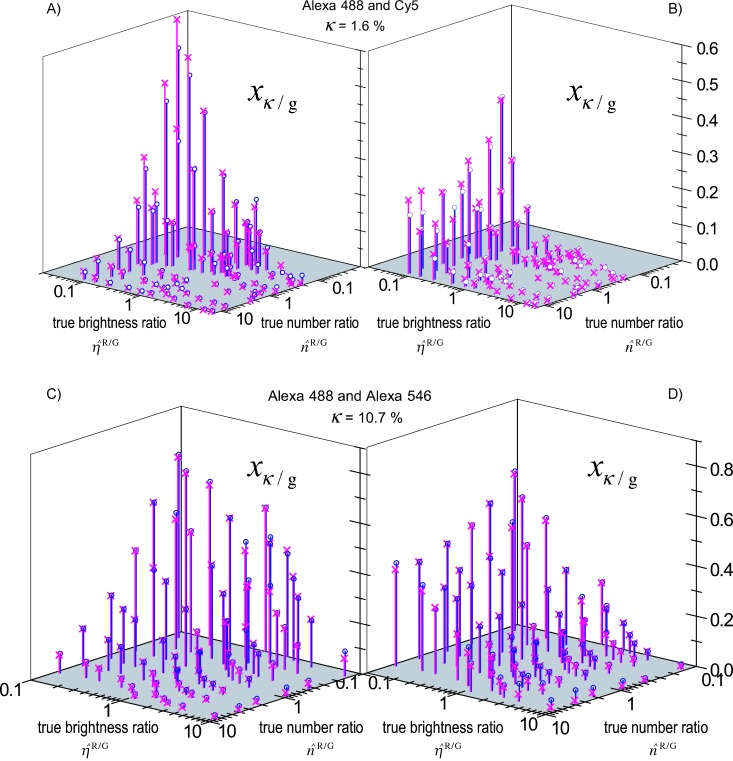
Cross-talk artifact predictions using brightnesses and numbers obtained in a separate measurement. Artifactual relative cross-correlation obtained from mixtures of green and red dye molecules in the absence of double-labeled species. Theoretical values predicted as in Figure 1 B from brightness and number ratios and the indicated *κ* value are denoted by crosses; experimentally determined relative cross-correlations are denoted by open circles. Data points have been slightly shifted in the horizontal plane to allow visualization of both experimental and theoretical values in a single diagram. A, B) Relative cross-correlations as a function of real brightness and number ratios ( 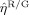 and 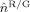). To determine these, separate measurements were performed in which the 488 nm laser was switched off. The prediction using the bleed-through ratio *κ*_cal_=1.6 % from the calibration measurement is mostly successful. At low red brightnesses, however, the artifactual cross-correlation is overestimated. The reason for this discrepancy is that the red brightness is underestimated from the separate measurements, in which the 488 nm laser does not contribute to the excitation of the red dye (Cy5), whereas it does so in the actual cross-correlation measurement. C, D) Relative cross-correlations as a function of real brightness and number ratios ( 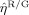 and 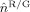) for a dye and filter combination with larger spectral cross-talk (Alexa 488, Alexa 546; *κ*_cal_=10.7 %).

As shown in Figure 1 C, simple substitutions of apparent red brightness and particle number from the actual measurement directly for the true brightness and particle number yield aberrant cross-talk predictions at low red brightness and low red particle number. The reason is that the red autocorrelation curve and thus the brightness determination are affected by cross-talk. One option to exclude cross-talk and determine the true number and brightness ratios 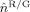 and 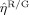 is to perform a separate measurement, in which the blue laser excitation is switched off to prevent excitation of the green dye and thereby cross-talk from the green dye into the red channel (Figure 2). However, fewer measurements are required and accuracy is improved if the relative artifactual cross-correlation is expressed in terms of the apparent number and brightness ratios 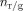 and 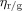 obtained directly from the actual measurement (Eqs. (34b) and (35b), derived in the Experimental Section)


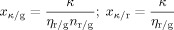


or, equivalently [Eqs. (34a) and (35a)]


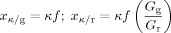


where *f*=*F*_g_/*F*_r_ is the apparent count-rate ratio of the green and the red detection channels.

Equation (34a) offers a particularly simple way to check for cross-talk during the experiment. The cross-correlation caused by cross-talk, *x*_*κ*/g_, is given by the product of the bleed-through ratio *κ* and the ratio *f* of the apparent (i.e. measured) count rates in the green and the red channel. These quantities are easily determined in the experiments. Furthermore, it is intuitively satisfying that the cross-talk artifact 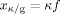
is equal to the fraction of the fluorescence signal in the red channel that comes from cross-talk as opposed to the true red fluorescence, because 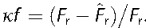


### Cross-Talk Correction

Use of the ratio of the cross-correlation amplitude to the green autocorrelation amplitude *x*_/g_, called *x* for short, permits rapid assessment of the cross-talk contribution as well as easy removal. As derived in the Experimental Section, the cross-talk-corrected cross-correlation 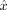 is given by [Eq. (6)]


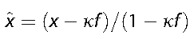


Equation (38) shows that the cross-talk-induced cross-correlation *κf* needs to be subtracted from the relative cross-correlation *x* and the remaining cross-correlation scaled up by 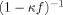. From the case of full binding, where both 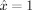 and 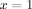 need to be fulfilled despite 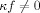, it is obvious that the subtraction of the cross-talk-induced cross-correlation does not suffice. A subsequent rescaling step is necessary.

### Cross-Talk Correction of Absolute Amplitudes

It is also possible to correct the absolute amplitudes by removing the cross-talk contribution according to Equations (27)–(29):


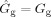



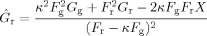



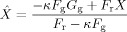


Note that Equation (29) can be rewritten as 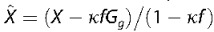. Dividing both sides by *G*_g_ returns Equation (38). The correction of the absolute cross-correlation amplitude *X* hence entails subtraction of 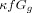, followed again by a rescaling step with 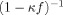.

Amplitude corrections for the more complicated case of bidirectional cross-talk are described in the Experimental Section.

### Experimental Validation

**Figure 3:**
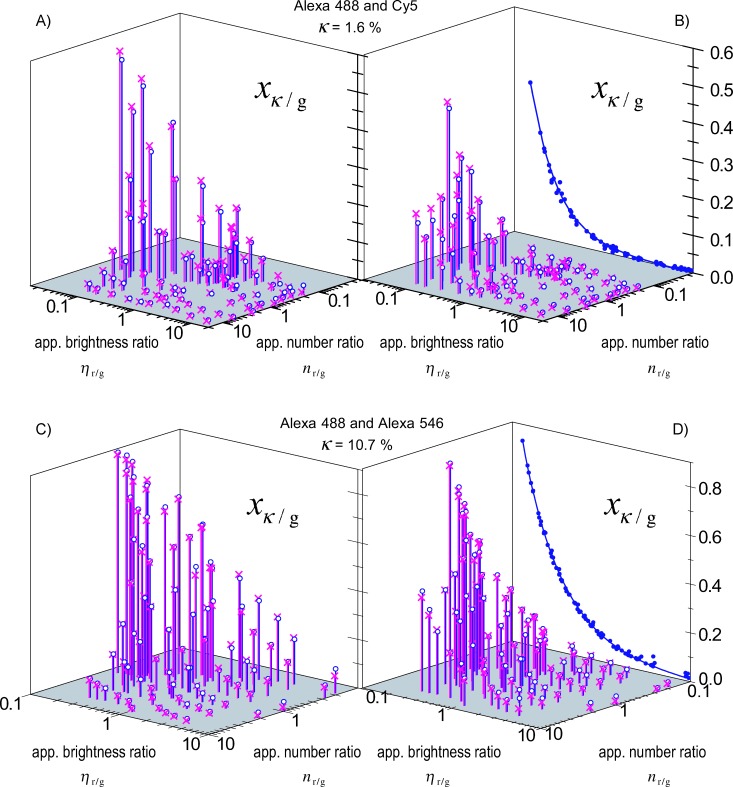
Cross-talk artifact predictions using apparent brightnesses and numbers directly from the measurement. A, B) Relative cross-correlations as a function of apparent brightness and number ratios 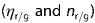, determined directly from the count rates and amplitudes obtained in the cross-correlation experiment. The calculation (predicted values, crosses) provides a reliable estimate of the artifactual relative cross-correlation (open circles). The projection of the experimental values in (B) fits a hyperbolic function, which confirms that *x*_*κ*/r_ is independent of 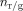 [Eq. (35b)]. The hyperbolic fit also allows an independent determination of the bleed-through ratio *κ*, yielding *κ*=1.5 %, which is close to the value of *κ*_cal_=1.6 % that was determined in the initial calibration [Eq. (3)] and was used for the cross-talk prediction. C, D) Relative cross-correlations as a function of apparent brightness and number ratios ( 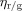 and 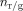) for a dye and filter combination with larger bleed-through (Alexa 488, Alexa 546; *κ*_cal_=10.7 %). The calculation provides very good estimates for the expected artifactual relative cross-correlation. Projection: from fitting Equation (13a), a bleed-through ratio of *κ*=10.8 % is recovered, which is close to the value of 10.7 % obtained by the simple calibration.

**Figure 4:**
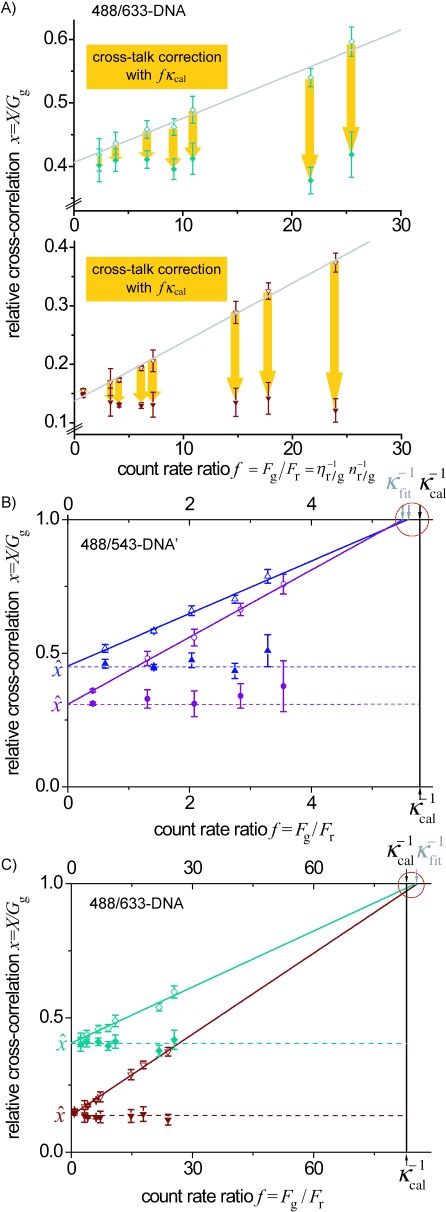
Effectiveness of cross-talk correction in the presence of green excess. A) Two samples with different proportions of double-labeled species (488/633-DNA) and red-labeled species (633-DNA) were prepared (top and bottom graphs). The binding degree with respect to the total amount of red particles, *θ*=*N*^RG^/*N*^R^, is given by the amplitude of the cross-correlation relative to the green autocorrelation, *x*=*X*/*G*_g_. Increasing amounts of extra green molecules (488-DNA) were added and dcFCCS measurements were performed after each addition. The apparent (i.e. measured) relative cross-correlation values were plotted versus the apparent green-to-red count-rate ratios. Error bars indicate single standard deviations of multiple measurements. The apparent relative cross-correlation increases solely due to cross-talk (open symbols). Each data point was independently cross-talk corrected by using Equation (38) and the bleed-through ratio from calibration, *κ*_cal_=1.2 % (filled symbols). B) The same procedure was applied to two different mixtures of 488/543-DNA′ and 543-DNA′. Increasing amounts of 488-DNA′ were added. The relative cross-correlation increases due to increasing cross-talk (open symbols). Each data point was cross-talk corrected using the bleed-through ratio from calibration, *κ*_cal_=17.3 % (filled symbols). Fitting a line to the apparent relative cross-correlation values provides an alternative way of obtaining the true relative cross-correlation, 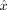, by finding the intersection with the ordinate axis (Eq. (4); indicated by the dashed lines). In addition, the intersections with the *x*=1 line yield *κ*_fit_^−1^ which is in reasonable agreement with *κ*_cal_^−1^. The value *κ*_cal_ from the initial calibration is slightly smaller than the bleed-through values obtained by fitting the data sets, *κ*_fit_, which is why the cross-correlation values corrected using *κ*_cal_ lie above the dashed lines. Errors increase with increasing *κf*. C) The data from (A) were plotted on a different scale, which allows the comparison of the values of *κ*_fit_ from the data sets with *κ*_cal_ from the initial calibration. They are in reasonably good agreement. For the 488/633 configuration, dcFCCS measurements tend to be limited by the usable range of count-rate ratios *f*. Errors remain rather small because of the small bleed-through ratio *κ*.

Since both FCCS samples and FCCS experimental conditions are usually nonideal, in particular with respect to sample labeling degree and the geometry of the detection volumes,^11^ cross-talk correction theory needed to be put to the experimental test. Samples containing no double-labeled species, that is, where the entire cross-correlation is due to cross-talk (Figure 1 C and Figures 2 and 3), as well as samples containing double-labeled species (Figure 4), were tested.

Figures 2 and 3 show that the cross-talk theory correctly predicts the relative cross-correlation due to cross-talk, based on a calibration value for *κ* and either separate measurements to determine the true brightnesses and particle numbers (Figure 2) or, more conveniently, the apparent brightness and particle number ratios (Figure 3). Experiments were performed on an almost completely separable dye pair (Alexa 488 and Cy5, Figure 2 A,B, Figure 3 A,B) and on a spectrally closer dye pair (Alexa 488 and Alexa 546, Figure 2 C,D, Figure 3 C,D).

Use of the apparent brightness and number ratios is not only faster, it also eliminates the systematic error that arises from cross-excitation: for the red dye, not only the desired laser line (here: 633 or 543 nm), but also the other laser line (here: 488 nm) contributes to its excitation and emission. If a separate measurement without the blue laser excitation is performed for the purpose of “switching off” the emission cross-talk from the green dye, the brightness of the red dye becomes underestimated. As a result, the cross-talk contribution can be overestimated in the regime of low red brightnesses.

### The Need for Cross-Talk Correction

How does the cross-talk affect the relative cross-correlation in samples that contain real double-labeled species? In the classical controlled cleavage experiment,^22^ the 1:1 stoichiometry of the labels in the starting material (*C*^R^=*C*^G^) simplifies the cross-talk handling. In the absence of brightness changes induced by quenching or fluorescence resonance energy transfer (FRET),^23^, ^24^ the green autocorrelation and the fluorescence leaking into the red channel are all constant ( 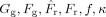 are constants). The fraction of double-labeled species constitutes the degree of association, *θ*(*t*)=*C*^RG^(*t*)/*C*^R^= 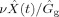 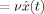, where *ν* is a proportionality constant that accounts for different effective detection volumes for the green channel and the cross-correlation.

According to Equation (33), which becomes 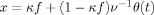, the apparent cross-correlation relative to the green autocorrelation, *x*, remains a linear function of the degree of association, *θ*(*t*). Cross-talk merely causes a constant offset.

However, in a titration experiment where, starting with the red-labeled species, more and more green-labeled species is added, the amount of fluorescence leaking into the red channel increases. In this case, cross-talk correction according to Equation (38) or Equations (27)–(29) is necessary.

### The Outcome of Cross-Talk Correction

To explore the effectiveness and the limitations of this cross-talk correction, a “binding” situation was artificially prepared by mixing double-labeled 488/633-DNA with red-labeled 633-DNA at an arbitrary ratio (Figure 4 A). Subsequently, the apparent relative cross-correlation was determined in a series of measurements after adding increasing amounts of green-labeled 488-DNA. According to Equation (1), the true cross-correlation relative to the green autocorrelation amplitude should not change upon the addition of green particles, because the numbers of double-labeled (*N*_RG_) and total red-labeled (*N*_R_) particles remain constant. However, cross-talk causes the apparent relative cross-correlation to rise with the addition of excess green molecules. As indicated by the arrows in Figure 4 A, each data point was cross-talk corrected according to Equation (38) using the bleed-through value *κ*_cal_ obtained from an initial calibration measurement (488/633 system: *κ*_cal_=1.2 %; 488/543 system: *κ*_cal_=17.3 %): Cross-talk correction successfully returns an approximately constant value for the true relative cross-correlation 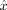. The experiment was repeated starting with a different proportion of double-labeled and red-labeled DNA (Figure 4 A, bottom). Likewise, two series of measurements were performed using a spectrally less separated dye pair (488/543-DNA′, Figure 4 B).

### Accuracy of Bleed-Through Determination

Rearranging Equation (33) yields [Eq. (10)]


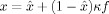
(4)

which demonstrates that the dependence of the apparent relative cross-correlation *x* on the apparent count-rate ratio *f* is linear and the intercept with the ordinate axis reflects the true relative cross-correlation in the absence of cross-talk, 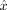.

Linear fits of the experimental series hence provide another way of determining a value for the true cross-correlation 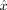 and the bleed-through *κ*. For the 488/633 configuration, the two series yield bleed-through values of *κ*_fit_=1.17 and 1.16 %, which is in good agreement with the value from the initial calibration, *κ*_cal_=1.2 %. For the 488/543 configuration, the two series yield values of *κ*_fit_=17.7 and 17.5 %, which agrees well with the initial calibration, *κ*_cal_=17.3 %.

As shown in the Experimental Section [Eqs. (40) and (33)], the apparent relative cross-correlation *x* does not increase indefinitely, but approaches 1 as *f* approaches 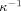 (rendering cross-talk correction impossible). The intersections of the fitted lines with the *x*=1 line thus occur at 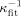 (Figure 4 B,C), very close to the value from the initial calibration ( 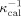
=(1.2 %)^−1^=83 for 488/633 and 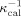
=(17.3 %)^−1^=5.78 for 488/543). A series of measurements at different green excess, such as in Figure 4 B and C, can thus be used as a more accurate way of determining *κ*. Yet, this approach is much more elaborate and time-consuming than the simple calibration based on Equation (3).

### Cross-Talk Correction Is Limited by *κf*

Importantly, the experiments in Figure 4 B and C illustrate the limitations to cross-talk correction. Errors sharply increase with increasing count-rate ratio *f* (Figure 4 B). According to Equation (38), the cross-talk-induced cross-correlation *κf* is subtracted from the relative cross-correlation *x* and the remaining cross-correlation is scaled up by (1−*κf*)^−1^, which also amplifies the measurement error by a factor of *a*=(1−*κf*)^−1^ [Eq. (39)]. The crucial quantity that governs cross-talk is the product *κf*. This is intuitively clear, because according to Equation (6), 
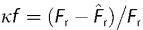
 represents the fraction of the red signal that comes from cross-talk and not from the truly red molecules. Hence, when only a small fraction of the red signal is a true signal from red molecules, the correction becomes inaccurate. For the 488/543 configuration, where the bleed-through is rather large (*κ*_cal_=0.173, Figure 4 B), the cross-talk-corrected relative cross-correlations start exhibiting large errors at moderate count-rate ratios (*f*≍3.5, that is, *κf*=0.6). Here, the rather large bleed-through *κ* restricts the usable range of count-rate ratios *f*. In contrast, the small bleed-through *κ* in the 488/633 configuration (*κ*_cal_=0.012) does not constitute a serious limiting factor, because measurements are restricted by the count-rate ratio itself, where the usable range is limited by the influence of noise at very low count rates *F*_r_ and nonlinear detector response at very high count rates *F*_g_. For the low bleed-through of *κ*_cal_=0.012, an extreme count-ate ratio of *f*=50 is required to reach *κf*=0.6.

## 3. Conclusions

The precise determination of the bleed-through ratio *κ* is important for any cross-talk correction and should be performed as a calibration measurement during each measurement session on a particular system of labeled molecules and optical setup.

Rough estimates of *κ* values are not sufficiently accurate for data correction, but are useful for predicting the approximate usable range of a dcFCCS application, as illustrated by the following example. The bleed-through, which is primarily governed by the selection of dyes and filters and is almost independent of laser intensity, has a value of *κ*=0.2 and a maximum value of *a*=2 for the error amplifying factor *a*=(1−*κf*)^−1^ is deemed acceptable, which corresponds to *κf*=0.5. In this case, count-rate ratios below *f*=2.5 will allow sufficiently accurate cross-talk correction. The count-rate ratio can be translated into true brightness and number ratios according to 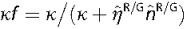 [Eq. (34)]. If dyes, filters, and laser intensities allow working with a favorable red-to-green brightness ratio of 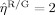, then a maximum green-to-red particle number ratio of 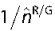 =10 will be permissible in the titration experiment.

Obtaining an estimate of *κ* for a given green dye and setup configuration is straightforward from a single dual-color count-rate measurement. Bleed-through values for the available filter configurations and typical dyes could thus be provided to users of commercial dcFCCS setups to guide their choices of fluorescent labels during experimental design. Nonetheless, precise determination of *κ* remains necessary for cross-talk correction.

Equation (34) shows that increasing the red-to-green brightness ratio 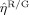 by increasing the laser power for the red dye and decreasing the laser power for the green dye offers a way of increasing the maximum permissible green-to red particle number ratio 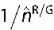 in an experiment. However, there are limitations to using a very low and a very high laser power. A low laser power means a low particle brightness, which directly affects the quality of the correlation curves.^9^, ^25^ Moreover, low count rates result in a greater uncertainty in the count-rate ratio *f* and thus in less reliability of the cross-talk correction [Eq. (39)]. Choosing excessively high laser powers causes photobleaching, which artifactually reduces the measured cross-correlation. The usable ranges of laser powers and brightnesses are determined for a given combination of the setup and type of sample by performing laser power series.^10^, ^26^

In this work, we have demonstrated that cross-talk in simple systems can be quantitatively corrected, without the need for specialized hardware or software. The ability to quickly assess and correct for cross-talk is of vital importance, because the most widely used commercial setups that combine FCS and confocal microscopy do not routinely offer fast alternating excitation schemes during FCS acquisition. Alternating excitation is only available in the confocal microscopy mode.

Cross-talk correction is simple, but it is restricted by combinations of the values of the bleed-through ratio, the brightness ratio, and the number ratio. The cross-talk correction scheme was validated herein by experiment only on a system with single, well-defined red and green brightnesses. The theoretical approach nonetheless is general and applicable also to systems with complex binding stoichiometries, complex labeling distributions, and fluorophore quenching, where brightness changes occur upon binding.^27^ In these more complicated cases, our cross-talk correction should be applied as the first step. As a second step, the cross-talk-corrected amplitudes will need to be analyzed taking into account the stoichiometry, labeling, and quenching situation.^20^, ^24^ Cross-talk correction and the interpretation of the correlation amplitudes can be performed independently of each other in this sequence. The correction scheme [Eqs. (5)–(33)] is based on total fluorescence count rates and does not make any assumptions about the interacting species with respect to their stoichiometry, brightness distributions, or changes in brightnesses due to FRET or quenching. Therefore, the correction scheme is independent of these effects. The only other quantity necessary for cross-talk correction, the cross-talk parameter *κ*, depends only on the spectra of dyes and the experimental setup (optical filters, etc.), but not on the system of interacting molecules that is being investigated. Therefore, cross-talk correction can be performed independently, as long as the spectral shape of the label that is causing the cross-talk and hence the cross-talk parameter *κ* does not change upon binding or with the degree of labeling.

Distinguishing dyes not only based on their emission spectra, but also based on their excitation spectra (by using alternating excitation schemes^28^, ^29^) or based on their lifetimes (by using time-correlated single photon counting^30^) expands the useful range of concentration ratios in dcFCCS applications with insufficiently separated labels. The quantitative cross-talk correction demonstrated herein is not only useful with setups that do not offer alternating excitation. It should also be useful when alternating excitation schemes are used on spectrally less separated dyes where the shorter-wavelength laser still excites both dyes, and emission from the longer-wavelength dye bleeds into the shorter-wavelength detection channel.

## Experimental Section

### Fluorescent samples

Alexa 488 hydrazide and Alexa 546 succinimidyl ester dyes were obtained from Molecular Probes (Invitrogen); Cy5 succinimidyl ester was from Amersham (GE Healthcare).

The double-labeled oligonucleotide Cy5-5′-CGT ACG CGG AAT ACT TCG ATT-3′-Alexa 488 and the single-labeled oligonucleotides Cy5-5′-CGT ACG CGG AAT ACT TCG ATT-3′ and 5′-CGT ACG CGG AAT ACT TCG ATT-3′-Alexa 488 were obtained PAGE-purified from IBA GmbH (Göttingen, Germany). Each type of labeled oligonucleotide was annealed to an unlabeled strand, 5′-TCG AAG TAT TCC GCG TAC GTT-3′, as previously described,^31^ yielding three samples which are designated by the matching laser excitation wavelengths: 488/633-DNA, 488-DNA, and 633-DNA.

The Alexa 488 labeled oligonucleotide (5′-TAT GTC TCT GAC TGC TCG AAT TCA CTA TCG GCC AGT GATT-3′-Alexa 488) and its complementary strand (5′-AAT CAC TGG CCG ATA GTG AAT TCG AGC AGT CAG AGA CATA-Alexa 546) were labeled and purified by Thomas Ohrt and Karin Crell.^31^ By annealing the Alexa 546- and the Alexa 488-labeled strands, the double-labeled 488/543-DNA′ sample was obtained. In addition, each strand was annealed to complementary unlabeled oligonucleotides, thereby yielding the 488-DNA′ and 543-DNA′ samples.

All DNA samples were formed into aliquots and stored at −20 °C. Samples were thawed, diluted, and mixed in phosphate-buffered saline (PBS) immediately before use.

### Dual-color FCCS

Dual-color fluorescence cross-correlation measurements were carried out on a ConfoCor2 setup (Carl Zeiss, Jena, Germany) with a 40× NA 1.2 water immersion C-Apochromat objective, as described in ref. 10, using the 488 nm excitation line of the argon ion laser and either the 543 nm (for Alexa 546) or 633 nm helium–neon laser (for Cy5). The 488/543 configuration had a 505 to 530 nm bandpass in the green detection channel, a 570 nm secondary dichroic, and a 585 to 615 nm bandpass in the red channel. The 488/633 configuration had a 505 to 550 nm bandpass in the green channel, a 635 nm secondary dichroic, and a 650 nm longpass in the red channel.

To examine the influence of particle brightness, the illumination powers were varied using the acousto-optical tunable filter. Particle numbers were adjusted by combining solutions of different concentrations. FCS curves were fitted to the model equation for one diffusional component and one blinking term using the ConfoCor2/ConfoCor3 software.^2^ Cross-talk-affected cross-correlation curves were fitted with a fixed value for the triplet blinking time, which was determined from the corresponding green autocorrelation curve.

### Theory

We follow the description of the influence of cross-talk on the correlation functions by Ricka et al.^18^ We start out with the general case of bidirectional cross-talk (Green dye into red channel and Red dye into green channel). For definitions of the parameters, see Table 1.

The apparent (i.e. measured) fluorescence in the green channel includes cross-talk of the red dye [Eq. (11)]


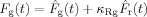
(5)

and vice versa [Eq. (12)]


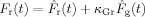
(6)

Using matrices [Eq. (13)]:


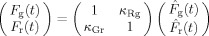
(7)

Inverting the matrix yields [Eq. (14)]


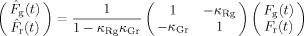
(8)

The non-normalized (nn) correlation functions are defined by [Eq. (15)]


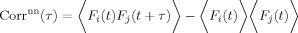
(9)

and the amplitudes at *τ*=0 by [Eq. (16)]


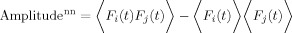
(10)


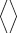
 denotes the temporal average, *i=j* for the case of autocorrelation and *i*≠*j* for cross-correlation.

The non-normalized correlation amplitudes are thus [Eqs. (11)–(13)]


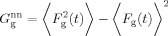
(11)


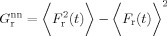
(12)


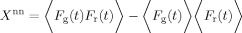
(13)

and [Eqs. (20)–(14)(16)]


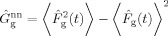
(14)


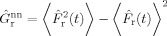
(15)


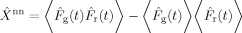
(16)

They relate to the normalized correlation functions as follows [Eqs. (17)–(23), (20)–(24)]


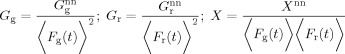
(17–19)


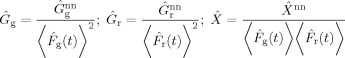
(20–22)

Inserting Equations (5) and (6) into Equations (11)–(13), and substituting the expressions from Equations (14)–(16) yields [Eqs. (23)–(25)]


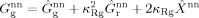
(23)


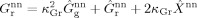
(24)


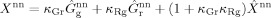
(25)

or, in matrix form [Eq. (26a)]


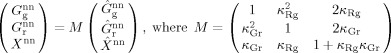
(26a)

The true (cross-talk-corrected) correlation amplitudes are calculated from the apparent (measured) ones by inverting the matrix *M*, which contains the cross-talk factors [Eq. (26b)]


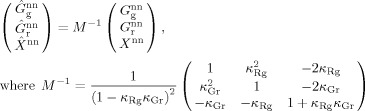
(26b)

Applying the following approach leads to cross-talk-corrected amplitudes in the general case of bidirectional cross-talk: the cross-talk parameters *κ*_Gr_ and *κ*_Rg_ have to be evaluated for the system by calibration measurements of pure green and pure red dye, respectively. Using these values, the cross-talk matrix *M* and its inverse *M*^−1^ are determined. The apparent correlation amplitudes ( 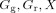) and average fluorescence count rates (*F*_g_, *F*_r_) of the actual, cross-talk-affected measurement are then used to calculate the non-normalized correlation amplitudes ( 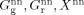) according to Equations (17)–(19). These are cross-talk-corrected by using Equation (26b). Finally, after determining the corrected fluorescence count rates ( 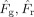) according to Equation (8), the corrected and normalized correlation amplitudes ( 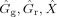) are calculated according to Equations (20)–(22).

A limiting case, where cross-talk correction must fail, occurs when the dyes are not distinguishable using the two detection channels. When 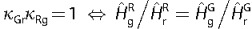, which means that the brightness ratio in the green and in the red channel is the same for both dyes, matrix *M* in Equation (26a) becomes singular and is not invertible.

In many applications, the cross-talk of the red dye into the green channel is zero (*κ*_Rg_=0), and only the cross-talk *κ*_Gr_=*κ* from the green dye into the red channel needs to be taken into account (Figure 1). In this unidirectional cross-talk case, the inverted cross-talk matrix *M*^−1^ simplifies to [Eq. (26c)]


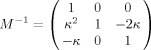
(26c)

The true, normalized correlation amplitudes expressed in terms of measurable quantities are then [Eqs. (27)–(29)]


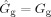
(27)


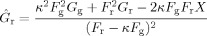
(28)


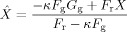
(29)

Vice versa, the apparent (measured) correlation amplitudes can be expressed in terms of the true correlation amplitudes by applying the special case of unidirectional cross-talk to Equation (26a) [see Eqs. (30)–(32)]


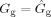
(30)


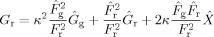
(31)


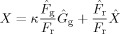
(32)

Because relative cross-correlation amplitudes are useful when thinking in terms of binding degrees [Eq. (1)], the cross-correlation is divided by the green (not cross-talk-affected) autocorrelation [Eq. (37)]


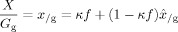
(33)

in short notation, 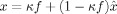, see Table 1 for parameter names.

In the absence of double-labeled species, the true cross-correlation is zero ( 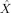, 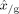
=0) and the remaining cross-correlation (*X*_*κ,*_ *x*_*κ*/g_) is entirely due to cross-talk. The cross-talk-induced cross-correlation can be calculated from *κ* in combination with either

a) the apparent (measured) count-rate ratio *f=F*_g_/*F*_r_,

b) the apparent brightness and number ratios, *η*_r/g_=*H*_r_/*H*_g_ and 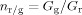, or

c) the true brightness and number ratios, using Eq. (6) [see Eqs. (34a–c)]


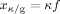
(34a)


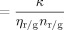
(34b)


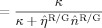
(34c)

Similar expressions can be derived for the cross-talk-induced cross-correlation divided by the red autocorrelation, where Equation (35a) follows directly from Equation (34a), Equation (35b) follows from Equation (35a), and Equation (35c) was derived from Equation (35b) using Equations (6) and (31) with 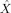
=0:


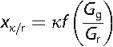
(35a)


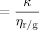
(35b)


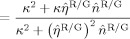
(35c)

Note that in an experiment where red and green particles have truly the same brightness and same number, that is, 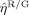
=1 and 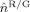
=1, the relative cross-correlations assume the value of *κ*, provided that the bleed-through *κ* is small [Eqs. (36a,b)]


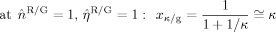
(36a)


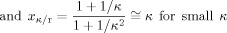
(36b)

The relative cross-correlation amplitudes in the case of the absence of truly double-labeled species [Eqs. (34), (35)] can also be computed for the more complicated situation of bidirectional cross-talk [from Eqs. (23)–(25) with Eqs. (20)–(22), (17)–(19), and (5)–(6)] [see Eqs. (37a,b)]


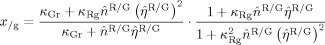
(37a)


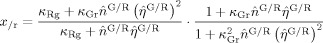
(37b)

where 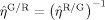 and 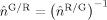.

**Figure 5:**
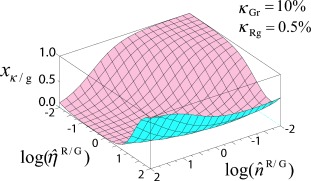
Simulation of the effect of bidirectional cross-talk on the relative cross-correlation amplitude 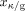 using Equation (37a). The bleed-through of the Green dye into the red channel *κ*_Gr_ in this example is 10 %. In contrast to Figure 1 B, this example assumes that there is a significant amount of “reverse” bleed-through from the Red dye into the green channel (*κ*_Rg_=0.5 %). With bidirectional bleed-through, the resulting cross-talk artifact becomes large not only when the green particles are in excess and brighter compared to the red, but also in the other extreme, when the red particles are in excess and brighter than the green particles. A relevant “reverse” bleed-through like in this simulation was not encountered in the experiments with Alexa 488/Alexa 546 and Alexa 488/Cy5 (Figure 2).

Note that the brightness ratio has an even stronger effect on the artifactual cross-correlation amplitudes than the number ratio (Figure 5).

Equations (37a) and (37b) simplify to Equations (34c) and (35c) when cross-talk is unidirectional (*κ*_Rg_=0).

In the typical experimental case, where cross-talk is unidirectional but both cross-talk and real double-labeled particles are present, the true (cross-talk-corrected) cross-correlation 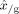 (called 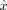 in short notation) is calculated from the apparent value 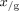 (*x* for short) using Equation (33) [see Eq. (048)]


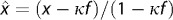
(38)

The cross-talk term *κf* has an adverse impact on measurement errors because, as opposed to the true relative cross-correlation values 0< 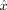
<1, the apparent relative cross-correlations occur in the narrower range of *κf*<*x*<1. Error propagation with respect to *x* yields [Eq. (49)]


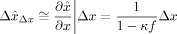
(39)

which means that the larger *κf*, the less certain the determination of the true cross-correlation 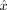. Uncertainties in the determination of *κ* (see Results and Discussion) and *f* further augment the overall error 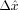.

Note that in the extreme case of a very large excess of green fluorescence and vanishing red particles, the count-rate ratio *f* approaches *κ*^−1^ (compare Figure 4) [Eq. (50)]


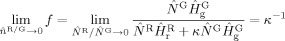
(40)

In this case, the red count rate stems entirely from cross-talk. Equation (33) shows that under these circumstances, the apparent (measured) cross-correlation *x* is 1, independently of the true cross-correlation 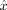. The true cross-correlation is no longer determinable, which is also evident from Equation (39).
